# Marine Ascomycetes Extract Antifungal Susceptibility against *Candida* spp. Isolates from Oral Candidiasis HIV/AIDS Patient: An *In Vitro* Study

**DOI:** 10.1055/s-0043-1768466

**Published:** 2024-02-22

**Authors:** Alexander Patera Nugraha, Mada Triandala Sibero, Kindi Farabi, Meircurius Dwi Condro Surboyo, Diah Savitri Ernawati, Tengku Natasha Eleena binti Tengku Ahmad Noor

**Affiliations:** 1 Department of Orthodontic, Faculty of Dental Medicine - Universitas Airlangga, Surabaya, Indonesia; 2 Immunology Study Programme, Postgraduate School, Universitas Airlangga, Surabaya, Indonesia; 3 Department of Marine Science, Fac. of Fisheries and Marine Science, Diponegoro University, Semarang, Indonesia; 4 Department of Chemistry, Faculty of Mathematics and Natural Sciences, Universitas Padjadjaran, Indonesia; 5 Department of Oral Medicine, Faculty of Dental Medicine - Universitas Airlangga, Surabaya, Indonesia; 6 Membership of Faculty of Dental Surgery, Royal Collage of Surgeon, Edinburgh University, United Kingdom

**Keywords:** Medicine, HIV/AIDS, Oral candidiasis, Marine Ascomycetes, Antifungal

## Abstract

**Objective** The etiology of oral candidiasis (OC) was *Candida albicans* , *C. krusei* , *C. dubliniensis* , *C. tropicalis* that are frequently found in human immunodeficiency virus/ acquired immunodeficiency syndrome (HIV/AIDS) patients. Marine ascomycetes (MA) have been widely reported as an important producer of various antibiotic compounds. However, there is limited study of antifungal compounds from MA against *Candida* species. The aim of this study was to investigate the antifungal susceptibility of MA against Candida spp. isolates from OC HIV/AIDS patient.

**Materials and Methods** *Trichoderma sp* . is a sponge-associated fungus collected from Karimunjawa National Park, Central Java, Indonesia. The validation of *C. albicans, C. krusei, C. dubliniensis, C. tropicalis* . was done by ChromAgar. This study was true experimental with post-test only control group design; the sample was four replications for each group. Nystatin administration (K +), the golden standard antifungal drug, was used. The minimum fungicidal concentration (MFC), minimum inhibitory concentration (MIC), and diffusion zone methods were done. Analysis of variance difference test, and post-hoc Tukey's honest significant different were done to analyze the significant different between groups ( *p* ≤ 0.05).

**Results** The MFC and MIC of MA against *C. albicans, C. krusei, C. dubliniensis* , and *C. tropicalis* were found at 12.5%. In addition, the greatest diffusion zone of MA against *C. albicans, C. krusei, C. dubliniensis* , and *C. tropicalis* was found at 12.5%. There is no appreciable difference in antifungal activity between K + and 12.5% of MA extract ( *p* ≥ 0.05).

**Conclusion** Concentration of 12.5% MA extract has antifungal susceptibility against *Candida spp* . isolates from OC HIV/AIDS patient.

## Introduction

The most prevalent oral symptom of the human immunodeficiency virus/ acquired immunodeficiency syndrome (HIV/AIDS) with low count of cluster differentiation 4 (CD4) in adults or children is oral candidiasis (OC), which can affect the tongue and other oral mucosal locations. 1 2 As the viral disease progresses, it has been observed that nearly all HIV-infected individuals have a *Candida* colonization and that up to 90 to 95% of them acquire clinical lesions. An earlier study performed in Dr. Soetomo, Surabaya, East Java, Indonesia, discovered that 68 patients out of the 88 adult HIV/AIDS patients exhibited clinical features of OC. 1 Additionally, out of a total of 28 HIV-positive pediatric patients, OC was discovered in 16 (57.14%) of them in an earlier study. 2 Several OC manifestations, including pseudomembranous candidiasis, acute and chronic erythematous candidiasis, and chronic hyperplastic candidiasis, can occur in HIV/AIDS patients. 3 Linear gingival erythema and oral hairy leukoplakia are two conditions that resemble the clinical sign of OC. 4 5 Immunosuppressive condition in HIV/AIDS patient may lead to decrease in oral mucosal immunity that gives the chance of normal flora in the oral cavity to become opportunistic. Dental caries and periodontal disease also can frequently be found in HIV/AIDS patient. 6 7 *Candida* species are opportunistic infections that affect people with compromised immune systems and immunosuppression. 1 2 A fungal overgrowth on the tissues and, occasionally, the appearance of a white flake on the mucosal areas describe this illness. 3 The most common cause of OC is *Candida albicans* . This species is frequently cited as the cause of oral thrush. 8 9 10 However, prior previous studies identified a number of other species, including *C. glabrata* , *C. dubliniensis, C. krusei* , and *C. tropicalis* , as the culprits of candidiasis at Dr. Soetomo General Hospital, Surabaya, East Java, Indonesia. These fungal infection causes a severe systemic illness called candidemia. 1 2

Furthermore, candidiasis will have a higher negative impact for AIDS patients. Because of their weaker immune systems, those with HIV/AIDS are more likely to develop OC. 11 12 Due to *Candida* species' tolerance to antifungal medication, this is made worse condition in HIV/AIDS patients. 13 14 15 Additionally, in order to defend themselves, Candida spp. create biofilm that increases their resistance to antifungal drug. 16 17

According to several researches, infections including coronavirus disease-2019 and multidrug resistant (MDR)-tuberculosis can coinfect people with *Candida* spp. This coinfection will increase the duration of the medicine and raise the mortality rate. 18 19 Additionally, recent research has shown that xerostomia radiation makes patients with head and neck cancer more susceptible to OC infection. 20 It is necessary to investigate for novel antifungal compounds due to the decreasing efficacy of medications and their negative effects. 21 The inability of current antifungal medications to treat OC in HIV/AIDS patients as a result of drug resistance has sparked basic research to develop novel antifungal medicines and HIV/AIDS medicine. 3 22

The sponge-associated microorganisms are renowned for being among the top producers of antibacterial chemicals. Moreover, numerous antibiotics including averantin, nidurufin, citrinin, Chlorohydroaspyrones A and B are isolated from sponge-associated fungi, especially from phylum ascomycetes. 23 Therefore, marine ascomycetes (MA) from a specific niche were carried out to gather possible bioactive substances. The last 10 years have shown that MA developed a large number of distinctive bioactive molecules. 24 25 26 27 28

Indonesia, as an archipelagic and maritime country, is home to massive MA that exist as a companion microbe. A filter-feeding creature called a marine sponge deposits a lot of MA that produces antibacterial chemicals. 29 However, this work did not employ the *Candida* spp. isolates from OC HIV/AIDS patient or extract the pure lead compounds, despite the fact that previous study showed that various sponge-associated fungus from Indonesia shown anti-C. *albicans* activity. 30 Additionally, *Aspergillus* sp. LS78 linked with marine sponges effectively yielded a new aspericacids A with antifungal efficacy against non-MDR *C. albicans* . 31 As a result, it may indicated that sponge-associated MA may possessed excellent potential as a new source of novel antifungal drugs against *Candida* spp. to treat OC in HIV/AIDS patients. Furthermore, the aim of this study is to investigate the antifungal susceptibility of MA against *C. albicans, C. dubliniensis, C. krusei* , and *C. tropicalis* isolates from OC HIV/AIDS patients.

## Materials and Methods

### Ethical Clearance, Study Design, and Sample Selection

At the Faculty of Dental Medicine, Universitas Airlangga, Surabaya, *Candida* spp. was isolated from OC patients after receiving ethical approval and appointment number 681/HRECC.FODM/IX/2022. In this cross-sectional study, patients who had been tested for HIV/AIDS positive by means of polymerase chain reaction examination and low CD4 about less than 200 cells/mm ^3^ examined by means of flowcytometry were recruited. The patient's parents or guardians provided written informed consent and informed to consent form prior to the oral evaluation. Without knowing the patient's immunologic condition or whether they had undergone highly active antiretroviral therapy or not, trained oral medicine professionals performed an oral examination on each Indonesian patient.

The patient was examined while seated in a dental chair, with the aid of disposable dental mirrors, sterile gauze pads, and tongue blades, all under good illumination. According to European Commission-Clearinghouse clinical diagnostic criteria, OC was identified by its clinical characteristics as (1) pseudomembranous type, (2) erythematous type, and (3) angular cheilitis. An oral swab sample was used in a microbiological test to pinpoint the specific *Candida* spp. that was infecting the patient. HIV/AIDS patients who had OC at the time of the evaluation had oral swabs taken. These swabs were brought to the lab for culture and immediately submerged in Sabouraud dextrose liquid medium. Colony morphology on ChromAgar and microscopic morphology on slide culture were used to identify the species of *Candida* . 1 2

### Metabolite Production and Extraction

*Trichoderma sp.* (code:KJMT.FP 3.2) was isolated from an unidentified marine sponge from Karimunjawa National Park, Central Java, Indonesia. The seed culture was prepared in V-22 (1%, glucose 0.5%, NZ-case [Sigma- Aldrich, Co., LLC.] 0.3%, yeast extract [Kyokuto Pharmaceutical Industrial, Co., Ltd.] 0.2%, Tryptone [Difco Laboratories] 0.5%, K _2_ HPO _4_ 0.1%, MgSO _4_ •7H _2_ O 0.05%, and CaCO _3_ 0.3%, pH 7.0) broth for 3 days at 200 rpm and 30°C. For production culture, in total 1% (v/v) of the seed culture was transferred into A16 broth media (0.2% glucose, 1.5% Pharma media, 0.3% CaCO _3_ , 1% Diaion HP-20, pH 7.0.) for 7 days (200 rpm, 30°C). Afterward 1-butanol was added for extraction with ratio 1:1, then evaporated at 35 °C. 32

### Determination of MIC and MFC of the Lead Compounds

On Inorganic Salt Starch Agar number 4 agar and Ascomycetes Isolation Agar all sponge-associated MA will be grown for 7 days in order to be used in this screening. All pure lead compounds will be dissolved in dimethyl sulfoxide (DMSO) to reach concentration of 100, 75, 50, 25, 12.5, and 6.25%. The minimum inhibition concentration (MIC) assay will be conducted using microwell-dilution method in 96-well plate. Each well will be filled by 195 µL of Sabouraud dextrose broth (SDB) with the *C. albicans, C. krusei, C. dubliniensis,* and *C. tropicalis* (0.5 McFarland) and 5 µL of the diluted pure lead compound solution. Nystatin will be used as a positive control, DMSO 5 µL/plate as a negative control, and SDB without any additional substances as a blank. The plate will be incubated for 24 hours at 32 °C. After 24 hours of incubation, in total 10 µL of Resazurin will be added into each well to give blue/purple color then incubated for 2 hours. The color changes from blue/purple to bright yellow indicates the bacterial growth inhibition. The lowest concentration that able to inhibit the bacterial growth will be determined as the MIC. Further, in total 10 µL of SDB from concentration that able to inhibit the bacterial growth will be inoculated onto SDA and incubated for 24 h at 32 °C. The lowest concentration with no bacterial growth on Sabouraud dextrose agar (SDA) will be determined as MFC value. 33 34

### Screening of Antifungal Activity against *Candida* spp.

To find the probable isolate with antifungal activity, the agar plug technique will be used. MA with concentration of 25 and 12.5% was used for diffusion zone method. *C. albicans, C. krusei, C. dubliniensis,* and *C. tropicalis* shall be maintained on SDA for 24 hours before to the test. The pathogen shall be completely inoculated on SDA on the implementation day after being diluted in physiological salt solution to a turbidity of 0.5 McFarland. After that, the MA will be cut into circles and placed on the SDA that has received the vaccination. 33 After being incubated for 24 hours at 32 °C, the MA's presence in a clear zone denotes their antifungal activity.

### Statistical Analysis

The study data were then all compiled and evaluated both descriptively and inferentially. A bar chart showing the mean and standard deviation of the data is displayed. The statistical package for social science (SPSS) version 20.0 for Windows was used to analyze the data. This version included the normality and homogeneity test ( *p* > 0.05), analysis of variance difference test, and post-hoc Tukey's honest significant different (HSD) with a different significance value of *p* < 0.05 (IBM corporation, Illinois, Chicago, United States).

## Results

**Fig. 1:**
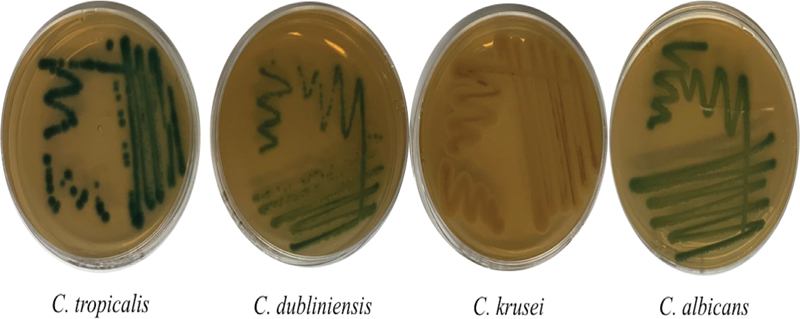
ChromAgar was used to characterize and identify the species of *Candida* spp. Human immunodeficiency virus/acquired immunodeficiency syndrome patient isolates revealed that there are four candida species such as *C. albicans* (light green color); *C. dubliniensis* (green to yellow color); *C. krusei* (pink to cream color) and *C. tropicalis* (dark green color).

From HIV/AIDS patient with low CD4 ^+^ with OC, the isolate of *Candida* spp. was obtained. ChromAgar was used to characterize and identify the species of *Candida* spp. HIV/AIDS patient isolates revealed that there are four *Candida* spp. such as *C. albicans* (light green color); *C. dubliniensis* (green to yellow color); *C. krusei* (pink to crème color); and *C. tropicalis* (dark green color Fig. 1 ).

**Fig. 2:**
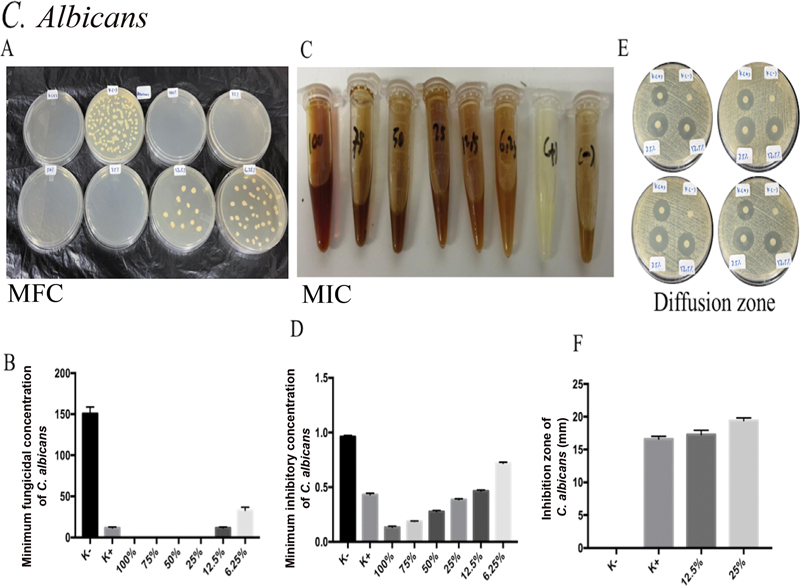
*Candida albicans* is the target of MA extract's antifungal action. ( **A** ) The MA extract's significant antifungal activity was seen in *C. albicans'* MIC values of 12.5, 25, 50, 75, and 100% following administration. ( **B** ) Following ingestion of MA extract, the MFC of *C. albicans* showed 12.5, 25, 50, 75, and 100% strong antifungal activity. After administration of MA extract, the inhibitory zone employing disk diffusion analysis on *C. albicans* revealed no appreciable difference in antifungal activity between K+ and 12.5% of MA extract. MA, marine *actinomycetes* ; MFC, minimum fungal concentration; MIC, minimum inhibitory concentration.

In this study, MA extract was found to have MIC and MFC and to be able to stop the development of the *Candida* species that are involved in OC in HIV/AIDS patients with low CD4+ levels: *C. albicans, C. dubliniensis, C. krusei* , and *C. tropicalis* . The nystatin treatment produced the highest levels of MIC and MFC of *C. albicans* , which were followed by MA extract concentrations of 100, 75, 50, 25, and 12.5% with significant differences ( *p* = 0.0001; *p* < 0.05; Fig. 2A–D ). Nystatin treatment, followed by MA extracts of 25 and 12.5%, produced the widest zone of *C. albicans* suppression ( Fig. 2E ). On the *C. albicans* inhibitory zone, there was no discernible difference between the treatment groups of K+ and 12.5% of MA extract ( Fig. 2F ).

**Fig. 3:**
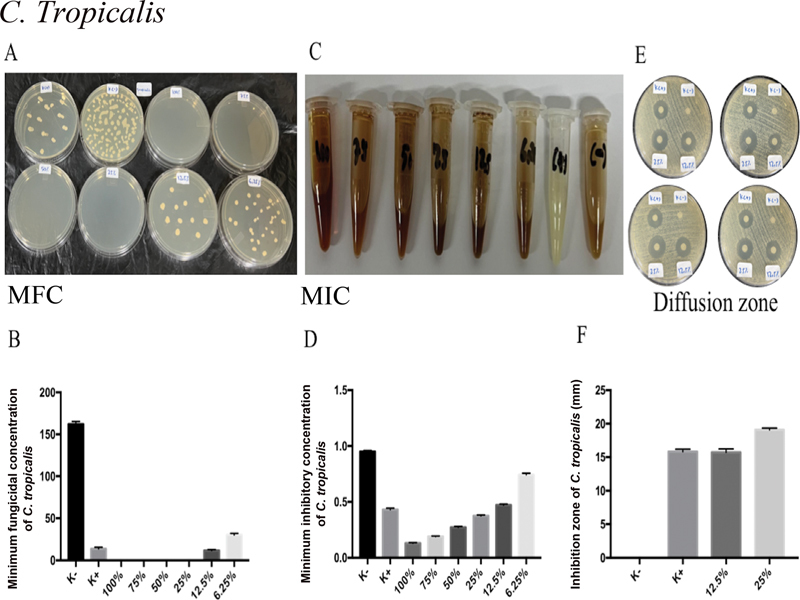
*Candida tropicalis* is the target of MA extract's antifungal action. ( **A** ) The MA extract's significant antifungal activity was seen in *C. tropicalis'* MIC values of 12.5, 25, 50, 75, and 100% following administration. ( **B** ) Following ingestion of MA extract, the MFC of *C. tropicalis* showed 12.5, 25, 50, 75, and 100% strong antifungal activity. After administration of MA extract, the inhibitory zone employing disk diffusion analysis on *C. tropicalis* revealed no appreciable difference in antifungal activity between K+ and 12.5% of MA extract. MA, marine *actinomycetes* ; MFC, minimum fungal concentration; MIC, minimum inhibitory concentration.

Nystatin treatment yielded the highest MIC and MFC of *C. tropicalis* , followed by MA extract concentrations of 100, 75, 50, 25, and 12.5%, with significant differences ( *p* = 0.0001; *p* < 0.05; Fig. 3A–D ). Nystatin treatment, followed by MA extracts of 25 and 12.5%, produced the widest zone of *C. tropicalis* inhibition ( Fig. 3E ). On the *C. tropicalis* inhibitory zone, there was no discernible difference between the treatment groups of K+ and 12.5% of MA extract ( Fig. 3F ).

**Fig. 4:**
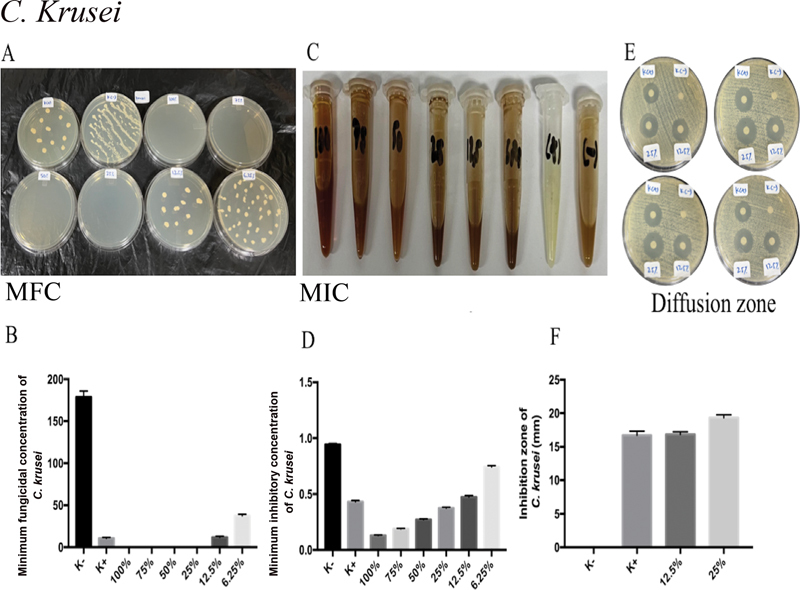
*Candida krusei* is the target of MA extract's antifungal action. ( **A** ) The MA extract's significant antifungal activity was seen in *C. krusei's* MIC values of 12.5, 25, 50, 75, and 100% following administration. ( **B** ) Following ingestion of MA extract, the MFC of *C. krusei* showed 12.5, 25, 50, 75, and 100% strong antifungal activity. After administration of MA extract, the inhibitory zone employing disk diffusion analysis on *C. krusei* revealed no appreciable difference in antifungal activity between K+ and 12.5% of MA extract. MA, marine *actinomycetes* ; MFC, minimum fungal concentration; MIC, minimum inhibitory concentration.

**Fig. 5:**
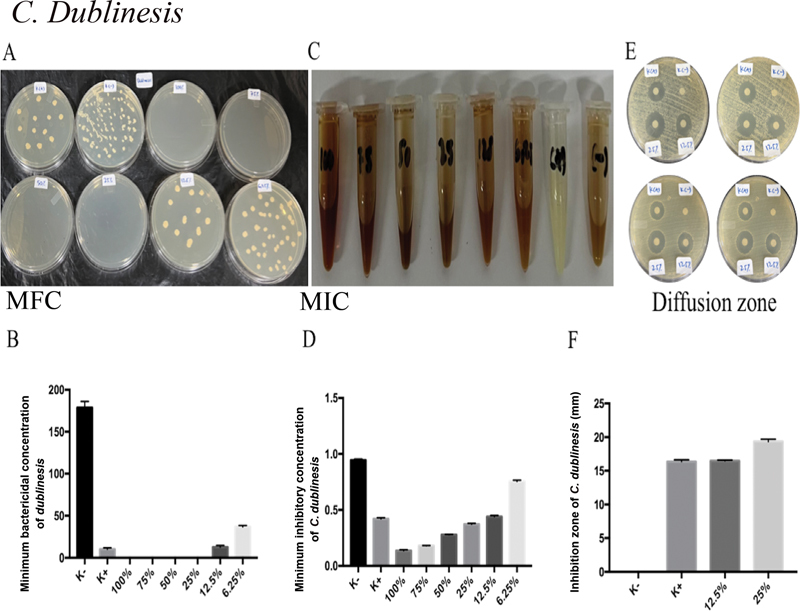
*Candida dubliniensis* is the target of MA extract's antifungal action. ( **A** ) The MA extract's significant antifungal activity was seen in *C. dubliniensis'* MIC values of 12.5, 25, 50, 75, and 100% following administration. ( **B** ) Following ingestion of MA extract, the MFC of *C. dubliniensis* showed 12.5, 25, 50, 75, and 100% strong antifungal activity. After administration of MA extract, the inhibitory zone employing disk diffusion analysis on *C. dubliniensis* revealed no appreciable difference in antifungal activity between K+ and 12.5% of MA extract. MA, marine *actinomycetes* ; MFC, minimum fungal concentration; MIC, minimum inhibitory concentration.

The MA extract of 100, 75, 50, 25, and 12.5% was shown to have significant differences in MIC and MFC of *C. krusei* (p = 0.0001; *p* < 0.05; Fig. 4A–D ) when compared to nystatin treatment. Following nystatin treatment, MA extracts of 25 and 12.5% were found to have the largest zones of C. *krusei* inhibition ( Fig. 4E ). On the *C. krusei* inhibitory zone, there was no statistically significant difference between K+ and 12.5% of MA extract as treatment groups ( Fig. 4F ). With a significant difference ( *p* = 0.0001; *p* < 0.05; Fig. 5A–D ), the highest MIC and MFC of *C. dubliniensis* were discovered in the nystatin treatment. Fig. 5E shows that nystatin treatment, followed by MA extracts of 25 and 12.5%, produced the widest zone of inhibition of *C. dubliniensis* . The inhibition zone of *C. dubliniensis* showed no significant change between the treatment groups of K+ and 12.5% of MA extract ( Fig. 5F ).

## Discussion

Human medicine has greatly benefited from the discovery and development of MA secondary metabolites. Even though there are hundreds of antibiotics and antifungals on the market right now, there is still a need and opportunity for the development of novel antimicrobials. Thus, there is a growing need for new antibiotics or alternative strategies to address antibiotic or antifungal resistance. 35

One of the major concerns to medicine today is the emergence of antibiotic-resistant bacterial infections, particularly those brought on by *Enterococcus faecium, Staphylococcus aureus, Klebsiella pneumoniae, Acinetobacter baumannii, Pseudomonas aeruginosa, and Enterobacter* species. 36 The antibiotics produced by MA that have been isolated from marine sponges have been shown to be effective against a variety of MDR bacteria. 37

If the aim is to identify clinical or veterinary medications, cytotoxicity to the patient should be ruled out when dealing with microbial crude extracts. From crude extracts generated from diverse species, methodologies have been established for the screening, isolation, identification, and characterization of important bioactive chemicals and secondary metabolites. 38 MA crude extracts also showed comparable findings, with the latter showing potential properties of therapies for antibiofilm development and quorum quenching. Therefore, the development of new antibiotics to treat MDR infectious diseases can come from MA that originated from Indonesia. 39 40

MA might be a source of antibiofilm agents of pathogens and other undesirable bacteria, when cellular development is not specifically controlled, other from by the limitation of quorum sensing. Since the selection pressure is “softer” with such antibiofilm agents, the evolution of resistance to antimicrobial products would be less likely. MA can be employed to prevent the development of biofilms and quorum sensing while avoiding the development of bacteria-evolved resistance, an issue frequently brought on by the use of antibiotics. 32 41 MA have been used in medicine as sources of secondary metabolites that have antibacterial, antifungal, anthelmintic, and anticancer properties. 29 The use of MA as probiotics has also been studied recently, while the majority of the study in this area is focused on applications in aquaculture and other aquatic settings. 42 The capabilities of additional MA may be investigated in this field, even if it is uncertain whether MA should be included in probiotics given to people. MA is expected to be as common in applied research as they are in the world's seas as more is learned about their special characteristics. 43

Among the worst dangers to human health across the world are infectious fungus infections. Fungal infections cause over 2 million fatalities annually worldwide, and the death toll is rising due to an increase in immunocompromised populations at risk. The introduction of infections that are resistant to first-line antifungal medications worsens the results. 43 44 The evolutionary history of fungus and mammals has impeded the development of novel antifungals, restricting treatment choices to medications due to low efficacy and/or harsh side effects. Like other antimicrobial leads, the majority of antifungal agents—including two of the three known antifungal classes—come from sources that are natural products. 45 46

This study investigated the antifungal susceptibility of MA against *C. albicans, C. dubliniensis, C. krusei,* and *C. tropicalis* isolates from OC HIV/AIDS patients. This study revealed that MA isolated from Karimunjawa National Park, Central Java, Indonesia has MFC and MIC against *C. albicans, C. krusei, C. dubliniensis, C. tropicalis* isolated from OC of HIV/AIDS patient at 12.5%. In addition, MA has greatest inhibition zone against C. *albicans, C. krusei, C. dubliniensis* , *C. tropicalis* was found at 12.5%. There is no appreciable difference in antifungal activity between nystatin and 12.5% of MA extract. There are several side effects of nystatin oral drop administration for OC treatment in HIV/AIDS patient such as nausea, vomiting, stomachache, headache, and diarrhea. 21 In addition, previous study also reports that there is Mangrove leaves ethanol extract ( *Aegiceras corniculatum* ) that had no antifungal efficacy against *C. albicans* isolated from HIV/AIDS with no significant difference in inhibitory zone ( *p* > 0.05). 47 Due to their effectiveness against MRSA and unique mode of action as antibacterials, MA will undoubtedly be the focus of intensive study. This is in addition to the rapidly expanding interest in natural products, particularly those originating from marine organisms. 37 48

In many Southeast Asian countries, medicinal plants are commonly used for antifungal activities. Among others, three plant species, that is, *S. aromaticum, C. citratus, C. xanthorrhiza,* are widely used for treating candidiasis in Indonesia, Malaysia, and Thailand. 49 One method for enhancing MA anti-Candida properties is by extracting their organic solvent, which releases associated bioactive chemicals. 50 51 Up to our knowledge, there have not been any previous reports on MIC and MFC values of MA isolated from Karimunjawa National Park, Central Java, Indonesia. To further improve MA extracts as the active ingredients in anti-Candida medications and dietary supplements, more research into the chemical components of the extracts as well as cytotoxicity tests on test subjects and mammalian cells is required.

## Conclusion

The OC of an HIV/AIDS patient was successfully isolated by our investigation from *C. albicans, C. krusei, C. tropicalis* , and *C. dubliniensis* . Nystatin antifungal resistance was found in *C. albicans* that was isolated from an HIV/AIDS patient's OC. Concentration of 12.5% MA extract has antifungal susceptibility against *Candida* Spp isolates from OC HIV/AIDS patients. Following the experimentation, numerous recommendations for future research have been made, including the need for enrichment and modified medium to minimize the isolation time and get more isolates of *Candida* spp. to expedite the investigation, a straightforward yet precise antifungal is required.
